# Modeling non-linear relationships in epidemiological data: The application and interpretation of spline models

**DOI:** 10.3389/fepid.2022.975380

**Published:** 2022-08-18

**Authors:** Noah A. Schuster, Judith J. M. Rijnhart, Jos W. R. Twisk, Martijn W. Heymans

**Affiliations:** ^1^Amsterdam UMC location Vrije Universiteit Amsterdam, Epidemiology and Data Science, Amsterdam, Netherlands; ^2^Amsterdam Public Health, Methodology, Amsterdam, Netherlands

**Keywords:** linear splines, restricted cubic splines, non-linearity, regression analysis, epidemiological methods, spline functions

## Abstract

**Objective:**

Traditional methods to deal with non-linearity in regression analysis often result in loss of information or compromised interpretability of the results. A recommended but underutilized method for modeling non-linear associations in regression models is spline functions. We explain spline functions in a non-mathematical way and illustrate the application and interpretation to an empirical data example.

**Methods:**

Using data from the Amsterdam Growth and Health Longitudinal Study, we examined the non-linear relationship between the sum of four skinfolds and VO_2_max, which are measures of body fat and cardiorespiratory fitness, respectively. We compared traditional methods (i.e., quadratic regression and categorization) to spline methods [1- and 3-knot linear spline (LSP) models and a 3-knot restricted cubic spline (RCS) model] in terms of the interpretability of the results and their explained variance ($r_{adj}^{2}$).

**Results:**

The spline models fitted the data better than the traditional methods. Increasing the number of knots in the LSP model increased the explained variance (from $r_{adj}^{2}=0.578$ for the 1-knot model to $r_{adj}^{2}=0.582$ for the 3-knot model). The RCS model fitted the data best ($r_{adj}^{2}=0.591$), but results in regression coefficients that are harder to interpret.

**Conclusion:**

Spline functions should be considered more often as they are flexible and can be applied in commonly used regression analysis. RCS regression is generally recommended for prediction research (i.e., to obtain the predicted outcome for a specific exposure value), whereas LSP regression is recommended if one is interested in the effects in a population.

## Introduction

In epidemiological research, regression analysis is often used to examine the association between an outcome and an exposure (1). A principal assumption of regression analysis is that the continuous exposure is linearly related to the outcome. In other words, a one-unit difference in the exposure is associated with a fixed difference in the outcome, regardless of the values of the exposure (2). However, linearity should not be assumed without assessing that the association is indeed linear (3–5). If the linearity assumption is violated and associations are estimated as linear nonetheless, then the effect estimate might not be a good representation of the true underlying effect and bias might be introduced. In order to obtain unbiased effects, the non-linear association requires explicit modeling. Failing to estimate a truly non-linear relationship as non-linear may lead to over- or underestimation of the exposure effect. However, it is important to note that the estimation of complex models may come at cost of increase uncertainty, especially in small samples. Therefore, in practice, one may want to consider the balance between model complexity and model uncertainty when choosing an appropriate method to model non-linear relationships.

There are different methods available to model non-linear associations. Simple methods such as polynomial regression (e.g., quadratic or cubic regression) and categorization of the exposure variable are widely used, largely due to historical precedent (6). With quadratic regression, for instance, the outcome is modeled as a quadratic function of the exposure (i.e., as a function of exposure *x* and the quadratic term *x*^2^) (2, 7, 8). Adding higher order terms (such as a quadratic term) to a basic linear function increases the flexibility of the model, but simultaneously complicates the interpretability of the results as the regression coefficients of the terms cannot be interpreted separately from each other.

With categorization, the exposure variable is grouped (e.g., based on percentile values) and subsequently analyzed as a categorical variable with one of the groups as the reference category. However, categorization is associated with multiple issues, such as loss of information, discontinuity in the estimated average outcome value when moving from one category to the other, and difficulties with comparing results across studies as the cut-off points may be data dependent (2, 6, 8–13). Filardo et al. found that study findings were inconsistent under different exposure categorization schemes identified in the literature, which suggests that the way the exposure is categorized may impact conclusions (14). This emphasizes the importance of correctly modeling non-linear relationships.

A different approach to model non-linear associations is the use of spline functions in the regression model (2, 3, 8, 11, 12, 15, 16). Spline functions are transformations of the continuous exposure variable and can be added to any regression analysis. They are available in different forms, such as simple linear spline (LSP) functions, more complex restricted cubic spline (RCS) functions and B-splines (2). Spline functions estimate exposure effects for specific intervals of the exposure variable and are subject to continuity restrictions (i.e., the interval functions meet at the common interval edges so that—in contrast to categorization—there are no jumps in the line at these points) (17). In this paper, we focus on LSP and RCS functions. LSP functions assume that the exposure effects within each interval follow a linear shape, but across the intervals the effect may be non-linear. Therefore, LSP functions are more flexible than simple linear regression and categorization. RCS functions assume that the exposure effects within each category are cubic functions, allowing for more flexibility than other methods.

Although spline functions are broadly accessible in the software packages commonly used by epidemiologists, they are not widely used (3, 18). Most papers published on spline functions present these as complex mathematical functions (15, 19, 20) and do not discuss their interpretation. This may be one of the reasons that researchers default to less optimal methods for estimating non-linear effects, such as quadratic terms and categorization.

The aim of this paper is to describe linear and restricted cubic spline functions in a step-by-step and non-mathematical manner, and to demonstrate the advantages of these methods over simple linear regression, quadratic regression and categorization using an empirical data example. First, we provide an introduction into spline regression and describe linear- and restricted cubic spline regression in the context of an empirical data example. Then, we illustrate the application of traditional methods and spline methods to model non-linear relationships to that same data example. Finally, we discuss the interpretation of the effect estimates from different methods and describe the context in which the use of LSP and RCS models may be relevant.

## Methods

### Example dataset

Spline functions will be explained by using an empirical data example from the Amsterdam Growth and Health Longitudinal Study (AGHLS). The AGHLS is an ongoing cohort study that was set up to examine the growth, health and lifestyle among teenagers (21). We use data from the third round of measurements, when the participants were 15 years old, because it contains a clear non-linear relationship.

**Figure 1:**
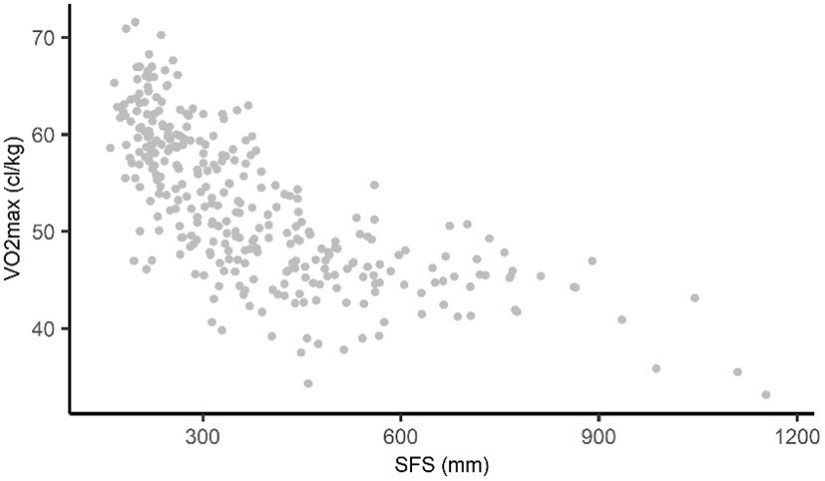
Non-linear relationship between SFS and VO_2_max in AGHLS data. SFS, sum of four skinfolds; AGHLS, Amsterdam Growth and Health Longitudinal Study.

Throughout this paper, we analyze the non-linear relationship between the sum of four skinfolds (SFS) and cardiorespiratory fitness (VO_2_max). SFS is an often used estimate of body fat and is calculated by summing the biceps-, triceps-, subscapular-, and suprailiac skinfolds (in millimeters) (22). VO_2_max is defined as the absolute maximal oxygen uptake in centiliter per kilogram bodyweight (21). The relationship between SFS and VO_2_max in our data is shown in Figure 1. Only subjects with complete data on both variables were included in the analysis (*n* = 315, 6 subjects were excluded because of incomplete data).

### Spline functions

Splines can be applied to any statistical model that linearly relates the exposure to the outcome, such as linear, logistic, and Cox regression. With spline models, the continuous independent variable is divided into multiple intervals, and for each interval the relationship between the exposure and outcome is estimated separately. The relationship between the exposure and the outcome in each interval can, for example, be estimated with a linear function (resulting in linear spline regression) or with a cubic function (resulting in cubic spline regression). The use of so-called *spline basis functions* makes it possible to estimate the relationship between the exposure and the outcome for each of the intervals in the same model. The values of the exposure based on which the intervals are created are referred to as *knots*. Thus, each knot defines the end of one interval and the start of the next. In 3-knot models, the exposure is divided into four intervals. Subsequently, for each interval the exposure effect is estimated, resulting in four spline coefficients. Corresponding confidence intervals can, for example, be calculated with the standard errors or be obtained by bootstrapping (23).

In general, a small number of knots (i.e., 3 to 5) is sufficient to model a non-linear relationship. If the sample size is large and the relationship that is studied changes quickly, then more knots might be required (2, 24, 25). Increasing the number of knots generally improves the fit of the model, but may also lead to overfitting of the model to the data. If that is the case, the fitted function does not only follow the main features of the data but also small and random fluctuations (2, 7, 25). Wand presents an overview of statistical methods for establishing the number of knots (26).

Often, the locations of the knots are pre-specified based on the quantiles of the independent variable. For 3-knot models, Harrell recommends knots at the 10th, 50th, and 90th percentile. For 4-knot models, they are recommended at the 5th, 35th, 65th, and 95th percentile (2). In some cases, knot locations are suggested by theory or by study design (e.g., an interrupted time series design). However, generally the fit of a spline model is more dependent on the number of knots than on the knot locations (25).

**Figure 2:**
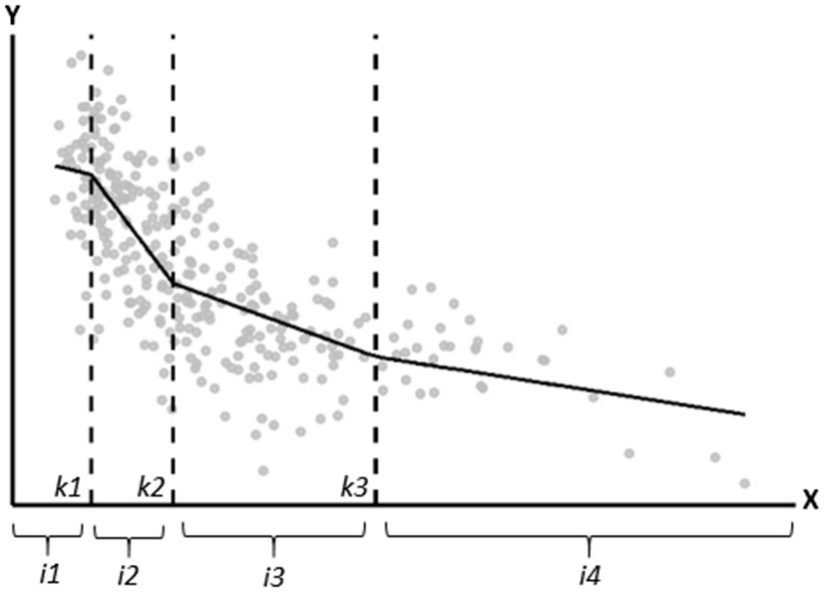
Graphical depiction of the important properties of a spline model. The gray points represent the observed data, and the black line is the fitted linear spline model. The vertical dotted lines represent the knots (located at *k1, k2*, and *k3*). *i1, i2, i3*, and *i4* represent the four intervals for which the exposure effect is estimated.

In this paper, for illustrational purposes, we demonstrate 1- and 3-knot linear spline models and a 3-knot restricted cubic spline model using the knot locations recommended by Harrell. Figure 2 shows the most important properties of a spline model. The gray points in Figure 2 represent the observed data, and the black line is the fitted 3-knot linear spline model. The vertical dotted lines represent the three knots (labeled as *k*1, *k*2, and *k*3) and the lines in between the knots represent the estimated exposure effect for the four intervals between the knots. Spline models are based on continuity restrictions, which ensures that the line is smooth at the knots. For example, the line for the first interval is smoothly connected to the line of the second interval, and the line of the second interval is smoothly connected to the line of the third interval, etcetera. An interactive visualization of LSP and RCS models and the influence of the continuity restrictions, number of knots and location of knots on the estimated line can be found elsewhere (27, 28).

### Linear spline models

In the 1-knot LSP model, the knot is located at the 50th percentile (*SFS* = 330 *mm*). The corresponding linear spline model is


(1) $$\begin{array}{r} VO2max=\;\beta_{0}+\beta_{1}\;*\;SFS+\beta_{2}^{*}\;*\;{\left(SFS-330\right)}_{+}+\varepsilon \end{array}$$

where β_0_ represents the intercept and ε represents an error term. To provide valid inference via e.g., confidence intervals for coefficients, it is assumed that the error terms for each observation are uncorrelated and follow a Gaussian distribution with expected value of zero. The term (*SFS*−330)_+_ represents the *spline basis function*. This function is assigned a value of zero when *SFS*−330 ≤ 0. Because of this, coefficient β_1_ represents the exposure effect estimate for individuals whose SFS is ≤330 mm. Coefficient $\beta_{2}^{*}$ represents the difference in the effect estimates between the individuals whose SFS is ≤330 mm and those whose SFS is >330 mm. Thus, for individuals whose SFS is >330 mm, their exposure effect estimate is represented by $\beta_{1}+\beta_{2}^{*}$. The 95% confidence interval corresponding to $\beta_{2}^{*}$ can be used to assess whether the slopes for the two intervals of SFS are statistically significantly different.

In the 3-knot LSP model, the knots are located at the 10th, 50th, and 90th percentiles, i.e., at SFS = 212, 330, and 621.4 mm, respectively. The corresponding LSP model is


(2) $$\begin{array}{l} VO2max=\beta_{0}+\beta_{1}\;*\;SFS+\beta_{2}^{*}\;*\;{\left(SFS-212\right)}_{+}\\ \;+\beta_{3}^{*}\;*\;{\left(SFS-330\right)}_{+}+\beta_{4}^{*}\;*\;{\left(SFS-621.4\right)}_{+}+\varepsilon \end{array}$$

In Equation 2, spline coefficient $\beta_{2}^{*}$ is only used whenever an individuals' SFS value is larger than 212, otherwise it is multiplied by zero and thus plays no role in the equation. For coefficient $\beta_{3}^{*}$ this is for *SFS*>330 and for coefficient $\beta_{4}^{*}$ this is for *SFS* > 621.4, respectively. Thus, coefficient β_1_ represents the exposure effect estimate for individuals whose SFS is ≤212 mm, while $\beta_{1}+\beta_{2}^{*}$ represents the effect estimate for individuals whose SFS is >212 and ≤330 mm. The exposure effect estimates for individuals in the third and fourth interval (i.e., individuals whose SFS is >330 mm and ≤621.4 mm, and individuals whose SFS is >621.4 mm) are represented by $\beta_{1}+\beta_{2}^{*}+\beta_{3}^{*}$ and $\beta_{1}+\beta_{2}^{*}+\beta_{3}^{*}+\beta_{4}^{*}$, respectively.

For both the 1- and 3-knot LSP models, fitting the spline models is straightforward once the spline basis functions have been established. Appendix A contains a step by step description of how to estimate these models, including R software code.

### Restricted cubic spline models

Although LSP models can approximate many relationships, they do not draw smooth lines and do not fit highly curved relationships well. This can be resolved by fitting a cubic spline model, which joins smoothly at the knot locations because the slopes are restricted to be equal at the boundaries (8). To improve the performance of the spline model in the tails of the exposure variable, where little data is located, additional constraints are imposed in *restricted* cubic spline models. In RCS models, the spline functions are linear in the tails (i.e., before the first and after the last knot) (2, 29). Whereas, in LSP models each interval is represented by a spline basis function, in RCS models *k*−2 spline variables are fitted, where *k* is the number of knots. Thus, in a 3-knot restricted spline function, a single spline basis function is fitted (Equation 3).


(3) $$\begin{array}{r} VO2max=\;\beta_{0}+\beta_{1}\;*\;SFS+\beta_{2}^{†}\;*\;SFS_{2}^{†}+\varepsilon \end{array}$$

where $SFS_{2}^{†}$ and $\beta_{2}^{†}$ represent the spline basis function and corresponding cubic spline coefficient (2). Each participant's value for the spline basis function is estimated as a function of the observed exposure value and the knot locations (i.e., SFS = 212, 330, and 621.4, respectively). The exact formula with which spline basis function $SFS_{2}^{†}$ is calculated is presented in Appendix B. Equation 3 can also be expressed as Equation 4, which contains the interval functions and has the same form as the 3-knot LSP model. The only difference between the LSP and RCS models is that for RCS regression all spline basis functions are raised to the power of three:


(4) $$\begin{array}{l} VO2max=\;\beta_{0}+\beta_{1}\;*\;SFS+\beta_{2}^{*}\;*\;{\left(SFS-212\right)}_{+}^{3}\\ \;+\beta_{3}^{*}\;*\;{\left(SFS-330\right)}_{+}^{3}+\beta_{4}^{*}\;*\;{\left(SFS-621.4\right)}_{+}^{3}\#\;+\varepsilon \end{array}$$

Equation 5 to 7 can be used to convert cubic spline coefficient $\beta_{2}^{†}$ into regression coefficients for each of the intervals:


(5) $$\begin{array}{r} \beta_{2}^{*}=\frac{\beta_{2}^{†}}{{\left(621.4-212\right)}^{2}} \end{array}$$

(6) $$\begin{array}{r} \beta_{3}^{*}=\frac{\beta_{2}^{*}*\left(212-621.4\right)}{\left(621.4-330\right)} \end{array}$$

(7) $$\begin{array}{r} \beta_{4}^{*}=\frac{\beta_{2}^{*}*\left(212-330\right)}{\left(330-621.4\right)} \end{array}$$

In Equation 5, $\beta_{2}^{*}$ represents the coefficient for the interval between the first and the second knot and $\beta_{2}^{†}$ is the cubic spline basis function coefficient from Equation 3. In Equation 6, $\beta_{3}^{*}$ represents the coefficient for the interval between the second and third knot and $\beta_{2}^{*}$ is the regression coefficient from Equation 4. In Equation 7, $\beta_{4}^{*}$ represents the coefficient for the interval after the third and $\beta_{2}^{*}$ is the regression coefficient from Equation 4. Subsequently, coefficients $\beta_{2}^{*}$, $\beta_{3}^{*}$ and $\beta_{4}^{*}$ can be plugged into Equation 4.

Like in quadratic regression, the exposure effect estimates differ across exposure values, which makes it less straightforward to interpret the coefficients from an RCS model.

## Results

We illustrate the interpretation and compare the performance of different methods to model non-linear relationships using the data example from the AGHLS. Table 1 presents the regression coefficients for each method. For the spline models, these regression coefficients are used to calculate the effects for each interval of SFS. These effects are presented under “interval coefficient.” Table 2 presents the adjusted *r*^2^ (i.e., the proportion of variance in VO_2_max explained by SFS) of each method (30).

**Table 1:** Regression- and interval coefficients for the relationship between VO_2_max and SFS derived from linear- and quadratic regression, categorization, 1- and 3-knot linear spline regression and 3-knot restricted cubic spline regression.

**Estimate**	**Regression coefficient**	**Interval coefficient**
**Linear regression**		
β_0_	64.0658	
β_1_	−0.0304	
**Quadratic regression**		
β_0_	73.2212	
β_1_	−0.0746	
β_2_	0.00004	
**Categorization**		
β_0_	60.1339	
β_1_	−4.9870	
β_2_	−10.0695	
β_3_	−15.1727	
**1-knot linear spline regression**		
β_0_	77.5648	
β_1_	−0.0810	*SFS* ≤330:−0.0810
$\beta_{2}^{*}$	0.0632	*SFS* >330:−0.0810+0.0632 = −0.0178
**3-knot linear spline regression**		
β_0_	64.1788	
β_1_	−0.0156	*SFS* ≤212:−0.0156
$\beta_{2}^{*}$	−0.0671	212 < *SFS* ≤330:−0.0156−0.0671 = −0.0827
$\beta_{3}^{*}$	0.0601	330 < *SFS* ≤621.4:−0.0156−0.0671+0.0601 = −0.0226
$\beta_{4}^{*}$	0.0128	*SFS*>621.4:−0.0156−0.0671+0.0601+0.0128 = −0.0098
**3-knot restricted cubic spline**		
β_0_	75.9306	
β_1_	−0.0738	
$\beta_{2}^{†}$	0.0740	$\beta_{2}^{*}$ : 0.0000004
		$\beta_{3}^{*}$ : −0.0000006
		$\beta_{4}^{*}$ : 0.0000002

$\beta_{2}^{*}$, $\beta_{3}^{*}$, and $\beta_{4}^{*}$ represent spline coefficients that correspond to spline basis functions.

*$\beta_{2}^{†}\;$* represents the cubic spline coefficient that corresponds to spline variable *$SFS_{2}^{†}$*.

**Table 2:** Explained variance of each model.

**Model**	**Adjusted *r*^2^**
Linear regression	0.487
Quadratic regression	0.558
Categorization	0.537
1-knot linear spline regression	0.578
3-knot linear spline regression	0.582
3-knot restricted cubic spline regression	0.591

**Figure 3:**
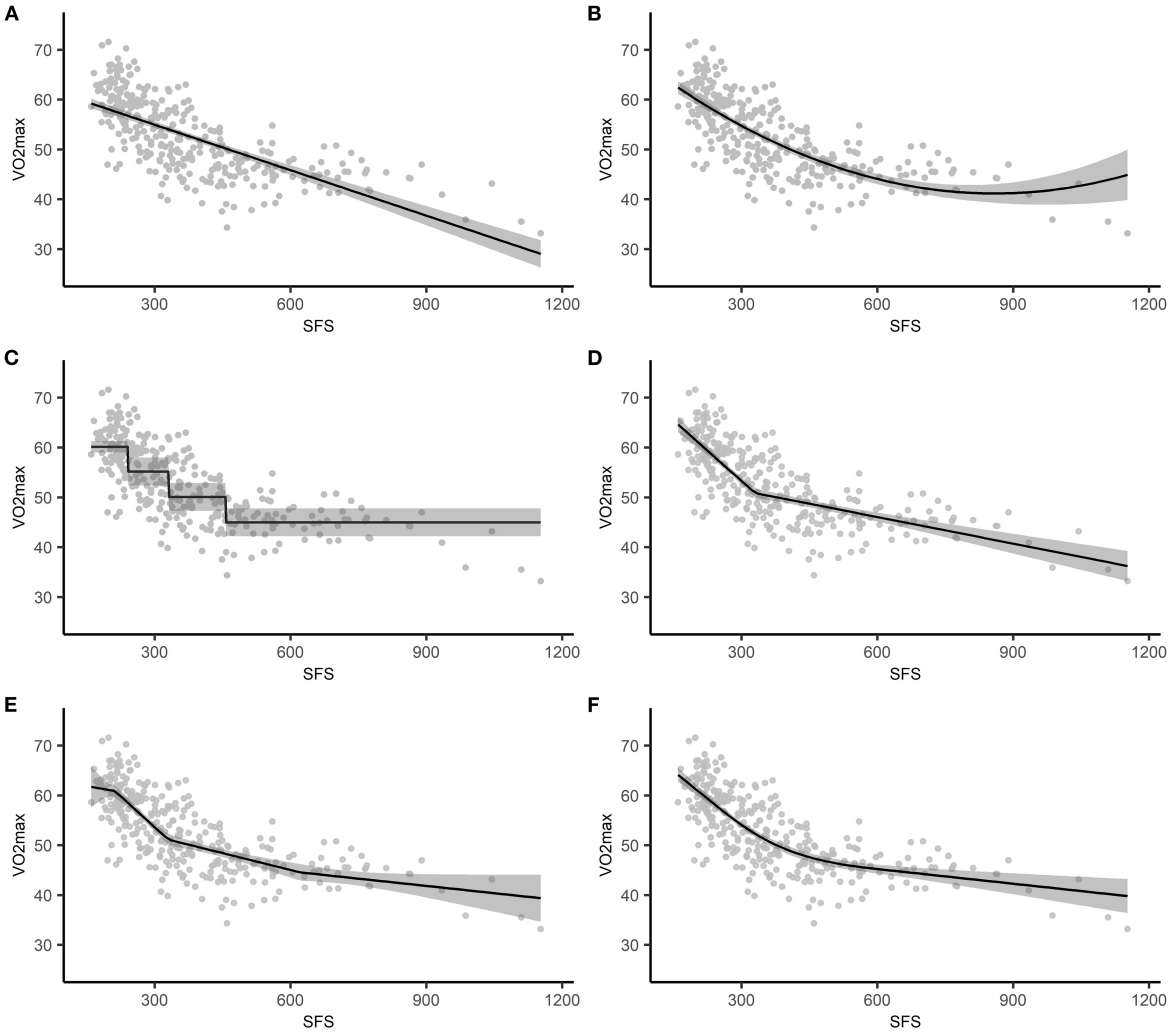
The estimated association between SFS and VO_2_max plotted against the observed values, with the shading representing the 95% confidence intervals based on standard errors. **(A)** simple linear regression, **(B)** polynomial regression, **(C)** categorization, **(D)** 1-knot LSP regression, **(E)** 3-knot LSP regression, **(F)** 3-knot RCS regression. SFS, sum or four skinfolds; LSP, linear spline; RCS, restricted cubic spline.

For illustrative purposes we first estimated a simple linear regression model. Linear regression fits a straight line to the data (Figure 3A) and assumes that the effect of the exposure on the outcome is the same for every value of the exposure. In our data, the exposure effect estimate was −0.0304, meaning that a 1 mm difference in SFS was associated with a 0.0304 cl/kg lower VO_2_max, regardless of the compared values of SFS. Naturally, this regression line was not a good representation of the relationship between SFS and VO_2_max, which was also reflected in the lowest explained variance ($r_{adj}^{2}=\;0.487$) of all estimated models.

### Quadratic regression

With quadratic regression, VO_2_max was estimated by SFS and the quadratic term *SFS*^2^. As shown in Figure 3B and reflected in the explained variance ($r_{adj}^{2}=0.558$), the quadratic model fitted the form of the relationship between SFS and VO_2_max quite well relative to the other models. However, the regression coefficients do not have a straightforward interpretation because the effect of SFS on VO_2_max is a function of both regression coefficients. That is, the effect of a one unit difference in SFS on VO_2_max differs across SFS. For example, the average difference in VO_2_max was −0.0506 cl/kg when SFS changed from 300 to 301 [i.e., (−0.0746 * 301+0.00004 * 301^2^)−(−0.0746 * 300+0.00004 * 300^2^)], while the average difference in VO_2_max was −0.0266 cl/kg when SFS changed from 600 to 601 [i.e., (−0.0746 * 601+0.00004 * 601^2^)−(−0.0746 * 600+0.00004 * 600^2^)]. Compared to simple linear regression (Figure 3A), the confidence interval for the line estimated using quadratic regression becomes wider for higher values of SFS (Figure 3B). This reflects the additional uncertainty in the effect estimates from quadratic regression for higher SFS values. However, the wider confidence interval does not affect the conclusion that SFS is associated with VO_2_ max.

### Categorization

We divided SFS into four intervals based on quartiles. Because we used the lowest quartile as the reference category, the intercept represented the mean VO_2_max in cl/kg for individuals in that interval. The regression coefficients represented the mean difference in VO_2_max between individuals in the lowest quartile and the other quartiles. For example, −4.9870 was the mean difference in VO_2_max in cl/kg between subjects in the first and second quartile. The explained variance was slightly lower relative to the other models ($r_{adj}^{2}=\;0.537$).

Figure 3C illustrates the assumed homogeneity within groups and the discontinuity in VO_2_max (i.e., the change in average VO_2_max value) when moving from one quartile to the next. For example, measures of SFS in the last quartile ranged between 458 and 1,153 mm, but all individuals had the same estimated VO_2_max of 44.9612 cl/kg (i.e., 60.1339−15.1727).

### 1-knot linear spline model

For individuals whose SFS was equal to or <330 mm, a 1 mm difference in SFS was associated with a 0.0810 cl/kg lower VO_2_max. The mean difference in the effect estimate between individuals in both intervals was 0.0632, meaning that for individuals whose SFS was >330 mm, a 1 mm difference in SFS was associated with a 0.0178 cl/kg lower VO_2_max (i.e., −0.0810+0.0632). Thus, for individuals whose SFS was >330 mm the association between SFS and VO_2_max was less strong than for individuals whose SFS was equal to or <330 mm. This is also illustrated in Figure 3D. The $r_{adj}^{2}$was 0.578. This indicates that the 1-knot linear spline model is a better fit to the data than both quadratic regression and categorization.

### 3-knot linear spline model

For individuals whose SFS was ≤212 mm, a 1 mm difference in SFS was associated with a 0.0156 cl/kg lower VO_2_max. For individuals whose SFS was between 213 and 330 mm, a 1 mm difference in SFS was associated with a 0.0827 cl/kg lower VO_2_max (i.e., −0.0156−0.0671). The interval coefficients for the other intervals can be found in Table 1.

Increasing the number of knots from 1 to 3 resulted in a slightly higher explained variance ($r_{adj}^{2}=0.578$ vs. $r_{adj}^{2}=0.582$, respectively). Furthermore, compared to simple linear regression (Figure 3A) and the 1-knot model (Figure 3D), the confidence interval for the line estimated using a 3-knot model becomes wider for higher values of SFS (Figure 3E). This reflects the additional uncertainty in the effect estimates from the 3-knot model for higher SFS values. However, the wider confidence interval based on the 3-knot model does not affect the conclusion that SFS is associated with VO_2_ max.

### 3-knot restricted cubic spline regression

Like with quadratic regression, separate interpretation of the coefficients is of no practical value with RCS regression, as the effect of SFS on VO_2_max is a function of multiple regression coefficients. For example, the average decrease in VO_2_max was 0.0644 cl/kg when SFS changed from 300 to 301 mm [i.e., (75.9306−0.0738 * 301+0.0000004 * (301−212)^3^)−(75.9306−0.0738 * 300+0.0000004 * (300−212)^3^)], while the average decrease in VO_2_max was 0.0244 cl/kg when SFS changed from 600 to 601 mm [i.e., (75.9306−0.0738 * 601+0.0000004 * (601−212)^3^−0.0000006 * (601−330)^3^) − (75.9306−0.0738 * 600+0.0000004 * (600−212)^3^−0.0000006 * (600−330)^3^)]. Figure 3F illustrates the restrictions (i.e., the function is linear for *SFS* ≤ 212 and *SFS* > 621.4) and shows that the model fits the data quite well. This is also reflected in the explained variance ($r_{adj}^{2}=\;0.591$).

## Discussion

The aim of this paper was to explain linear and restricted cubic spline functions in a step-by-step and non-mathematical manner and to demonstrate the advantages of these methods over simple linear regression, quadratic terms and categorization using an empirical data example. Although spline regression is easy to implement with most statistical programs, epidemiologists still often apply traditional methods (e.g., quadratic regression and categorization) to model non-linear relationships.

In the data example, the spline models resulted in higher explained variance than the traditional methods. Both categorization and spline regression divided the continuous exposure variable into intervals. Categorization only allows for variation between categories, so that the estimated outcome is the same for each individual in an interval regardless of their individual exposure value. This explains the stepwise pattern in Figure 3C. Spline regression, on the other hand, allows for variation between and within intervals. As a result, the regression line shifts between knot locations, and regression lines meet at the knot locations. Although polynomial regression is easy to model, it suffers from a lack of smoothness and can lead to implausible curvatures, in particular at the edges. Splines provide a good alternative as they control for this curvature via the continuity restrictions. In addition, RCS models are linear before and after the last knot. LSP models provide a good balance between modeling the non-linear association and providing results that are relatively easy to interpret. Furthermore, RCS models provide a flexible method for modeling the non-linearity of an association, but come at the cost of regression coefficients that are less easy to interpret than LSP models. In our data example, the explained variance in the LSP model and the RCS model were comparable. If one is interested in reporting the association between sum of four skinfolds and VO_2_max, then LSP models provide easier interpretations than the RCS models.

For both quadratic regression and RCS models, the increased complexity of the interpretation of the regression coefficients makes it less straightforward to summarize the exposure effect at the population level, because the exposure effect estimates differs in magnitude across exposure values. However, this is not necessarily a problem when the aim of a study is to make individual-level predictions of the outcome, as it remains relatively straightforward to compute the predicted outcome value for a specific exposure value using Equation 7 (8). Thus, in our data example, if one is interested in predicting VO_2_max based on specific values of the sum of four skinfolds, then RCS models may be preferred. Two things that might help with interpreting the results are the reporting of figures (such as Figure 3) and calculating the effect for a number of different exposure contrasts (i.e., the two exposure values that are being compared). The latter was done for the interpretation of the 3-knot RCS model, and showed that the decrease in VO_2_max was greater when SFS changed from 300 to 301 mm, then when it changed from 600 to 601 mm.

A strength of this paper are the non-mathematical explanations of LSP and RCS models. Although there are many other sources that describe spline models, most of these sources contain a high level of mathematical detail, which may discourage applied researchers from learning about these methods. In this paper, we tried to explained spline functions in a non-mathematical manner and in the context of an empirical data example. Furthermore, although we illustrated the application of spline models using cross-sectional data and within a linear regression context, the spline functions presented can be applied to all kinds of regression models, for example logistic and Cox regression. Further, they can also be used in longitudinal models such as generalized linear mixed models (GLMM) and generalized estimation equations (GEE).

Besides the methods discussed in the present paper, there are also other methods available that can be used to estimate non-linear associations. A method that we did not discuss is quadratic spline regression, in which the spline basis functions are quadratic functions. Although quadratic splines are often overlooked and not mentioned in known reference books (2), like cubic splines they result in smooth functions at the knot locations and can occur in restricted and unrestricted form. When the number of degrees of freedom are the same and the knots are located at comparable exposure values, restricted quadratic and cubic spline models might even yield similar results (31). SAS code for the estimation of restricted quadratic splines is provided by Howe et al. (31). Furthermore, we also did not discuss generalized additive models (GAMs), LOESS smoothing, penalized splines and fractional polynomials (32, 33), which are all capable of capturing non-linear relationships. However, these methods are relatively complicated and therefore, not much used in practice.

In this paper, we explained spline models based on a single exposure. However, in practice, researchers may want to adjust their association model for potential confounders of the exposure-outcome association. Most researchers are unaware that, if these confounders are continuous, then the linearity assumption also applies to these variables (34). Failing to explicitly model a non-linear confounder-outcome association may result in an under- or overestimation of the true exposure effect. Therefore, the linearity assumption should be assessed for each continuous confounder in a regression model, and splines can be applied when necessary.

Spline regression is easy to implement with most statistical software programs often used by epidemiologists. Table 3 contains a (non-exhaustive) overview of packages and macros available in different software programs. The analyses in this paper were conducted using the R programming language version 4.0.3 (35) and the “rms” package by Harrell (23). The R package “splines” is part of the basic distribution of R (29). Other frequently downloaded packages include “gss” (36) and “polspline” (37). An overview of spline methods and other R packages that may be used to fit spline models is presented elsewhere (29). In STATA, spline functions can be fitted using, among others, the STATA package “rmkspline” and the user-made package “RCsplines” (38). In SPSS, spline functions have to be fitted by hand and can be applied using the REGRESSION procedure. In SAS, the “effect” statement in “proc glimmix” provides an automated implementation for fitting splines. Documentation including syntax commands are available from the IBM support page (39) and the SAS Help Center (40).

**Table 3:** Spline regression options by software program.

**Software program**	**Packages/procedures**
R	rms, splines, gss, polspline
STATA	mkspline, RCsplines
SPSS	REGRESSION
SAS	TRANSREG

Although splines are easy to implement, they require certain choices to be made by the researcher. This concerns, for example, the number and location of the knots and the type of basis function (2). In addition, not all non-linear relations are “equally harmful” and the choice of spline model (e.g., linear or cubic) might depend on what's considered more important: LSP models might be used to model relations that only have a slight bend and that can be approximated by piecewise linear functions, whereas RCS might be used for maximum model accuracy. Another thing to consider is that some choices, such as increasing the number of knots, might introduce additional uncertainty to the model, especially in small samples. If the number of knots is too large, then the model overfits the data: it then describes the random error rather than the relationship between the variables. This affects the generalizability of the model outside of the data that it is based on (29). In our example, the confidence intervals were generally wider for more complex models, illustrating the additional model uncertainty introduced by more complex models. In some situations, the additional uncertainty might be a reason to use a more simple model.

## Conclusion

Spline functions should be considered more often in the analysis of non-linear relationships as they allow for more flexibility in estimating non-linear associations than traditional methods such as quadratic regression and categorization and can be used in all kinds of regression analyses. With RCS models the exposure effect estimates differ across exposure values, making them more suitable for prediction (i.e., to obtain the predicted outcome for a specific exposure value). If one is interested in the effects in a population, then LSP models are more suitable due to the straightforward interpretation of the regression coefficients.

## Data availability statement

The original contributions presented in the study are included in the article/Supplementary materials, further inquiries can be directed to the corresponding author/s.

## Ethics statement

The studies involving human participants were reviewed and approved by VU Medical Center. The patients/participants provided their written informed consent to participate in this study.

## Author contributions

NS and MH designed the study. NS performed the statistical analyses and drafted the manuscript. All authors contributed to data interpretation, critically revised the manuscript, and approved the final version of the manuscript.

## Conflict of interest

The authors declare that the research was conducted in the absence of any commercial or financial relationships that could be construed as a potential conflict of interest.

## Publisher's note

All claims expressed in this article are solely those of the authors and do not necessarily represent those of their affiliated organizations, or those of the publisher, the editors and the reviewers. Any product that may be evaluated in this article, or claim that may be made by its manufacturer, is not guaranteed or endorsed by the publisher.
