# Supplementary material for: Modeling non-linear relationships in epidemiological data: The application and interpretation of spline models
Source: Front Epidemiol. 2022 Aug 18;2:975380. doi: 10.3389/fepid.2022.975380 (PMC10910897; doi:10.3389/fepid.2022.975380)
Supplement: Supplementary file 1 [file Table_1.DOCX]

**Supplementary material**

**Appendix A**

Fitting 1-knot and 3-knot linear spline models (by hand) including R software code

library(rms)

attach(data)

*1-knot linear spline model*

1. Determine knot location according to 50^th^ percentile of SFS

t1 <- quantile(SFS, 0.50)

1. Compute SFS spline basis function xt1 and recode so that $SFS- t_{1}=0$if $SFS\leq t_{1}$

xt1 <- SFS – t1

xt1[xt1 <= 0] <- 0

1. Linear regression analysis including spline basis function xt1

ols(VO2max ~ SFS + xt1)

Automated implementation using the rms package: ols(VO2max ~ lsp(SFS, quantile(SFS, 0.5)), data = data)

*3-knot linear spline model*

1. Determine knot locations according to 10^th^, 50^th^ and 90^th^ percentile of SFS

t1 <- quantile(SFS, 0.10)

t2 <- quantile(SFS, 0.50)

t3 <- quantile(SFS, 0.90)

1. Compute SFS spline basis functions xt1, xt2 and xt3 and recode

xt1 <- SFS - t1

xt1[xt1 <= 0] <- 0

xt2 <- SFS - t2

xt2[xt2 <= 0] <- 0

xt3 <- SFS - t3

xt3[xt3 <= 0] <- 0

1. Linear regression analysis including spline basis functions xt1, xt2 and xt3

ols(VO2max ~ SFS + xt1 + xt2 + xt3)

Automated implementation using the rms package: ols(VO2max ~ lsp(SFS, quantile(SFS, c(0.1, 0.5, 0.9))), data = data)

**Appendix B**

Formula to calculate spline variable ${SFS}_{2}^{\dagger}$

$$\begin{aligned} {SFS}_{2}^{\dagger}=\frac{\left( SFS-212 \right)_{+}^{3}-\left( SFS-330 \right)_{+}^{3}*\frac{\left( 621.4-212 \right)}{\left( 621.4-330 \right)}+\left( SFS-621.4 \right)_{+}^{3}*\frac{\left( 330-212 \right)}{\left( 621.4-212 \right)}}{\left( 621.4-212 \right)^{2}} \#(B1) \\ \# \end{aligned}$$

where ${SFS}_{2}^{\dagger}$ is the spline variable and $SFS$ is the original exposure variable. Values 212, 330 and 621.4 represent the first, second and third knot location, respectively.

**Appendix C**

Fitting a 3-knot restricted cubic spline model (by hand) including R software

library(rms)

attach(data)

1. Determine knot locations according to 10^th^, 50^th^ and 90^th^ percentile of SFS

t1 <- quantile(SFS, 0.10)

t2 <- quantile(SFS, 0.50)

t3 <- quantile(SFS, 0.90)

1. Compute SFS spline basis functions xt1, xt2 and xt3 and recode

xt1 <- SFS - t1

xt1[xt1 <= 0] <- 0

xt2 <- SFS - t2

xt2[xt2 <= 0] <- 0

xt3 <- SFS - t3

xt3[xt3 <= 0] <- 0

1. Compute spline variable SFS_RCS using equation B1

SFS_RCS <- (xt1^3 - xt2^3 * ((t3 - t1)/(t3 - t2)) + xt3^3 * ((t2 - t1)/(t3 – t1)))/(t3 - t1)^2

1. Linear regression analysis including spline variable SFS_RCS

fit <- ols(VO2max ~ SFS + SFS_RCS)

1. Assign cubic spline coefficient, corresponding to spline variable SFS_RCS, to object csc

b0 <- fit$coefficients[1]

b1 <- fit$coefficients[2]

csc <- fit$coefficients[3]

1. Transform cubic spline coefficient csc into regression coefficients b2, b3 and b4 corresponding to spline basis functions xt1, xt2 and xt3 using equations 4 to 6

b2 <- csc/(t3 - t1)^2

b3 <- (b2 * (t1 - t3))/(t3 - t2)

b4 <- (b2 * (t1 - t2))/(t2 - t3)

The complete regression formula becomes

VO2max = b0 + b1 * SFS + b2 * xt1^3 + b3 * xt2^3 + b4 * xt3^3

Automated implementation using the rms package: ols(VO2max ~ rcs(SFS, 3), data = data)
